# Comparison of ex vivo harvested and in vitro cultured materials from *Echinococcus granulosus* by measuring expression levels of five genes putatively involved in the development and maturation of adult worms

**DOI:** 10.1007/s00436-016-5228-6

**Published:** 2016-08-12

**Authors:** Ebrahim Saedi Dezaki, Mohammad Mehdi Yaghoubi, Markus Spiliotis, Ghalia Boubaker, Elham Taheri, Pooya Ghaseminejad Almani, Farideh Tohidi, Majid Fasihi Harandi, Bruno Gottstein

**Affiliations:** 1 Research Center for Hydatid Disease in Iran, School of Medicine, Kerman University of Medical Sciences, Kerman, Iran; 2 Institute of Parasitology, Faculty of Medicine and Vetsuisse Faculty of the University of Bern, Bern, Switzerland; 3 Research Department of Biotechnology, Institute of Sciences and High Technology and Environmental Sciences, Graduate University of Advanced Technology, Kerman, Iran; 4 Department of Pathology, School of Medicine, Kerman University of Medical Sciences, Kerman, Iran

**Keywords:** *Echinococcus granulosus*, Cystic echinococcosis, Developmental genes, Real-time qPCR, In vitro cultivation

## Abstract

Parts of the natural life cycle of *Echinococcus granulosus* can be retraced in vitro such as the development of protoscoleces into semiadult worms with three or more proglottids, or the redifferentiation of in vitro cultured protoscoleces into metacestode-like cystic structures. Most in vitro generated samples share—at the microscopical level—high similarities with those naturally grown, but developmental differences have also been documented, such as missing egg production in in vitro grown adults or unusual bladder/vesicle formation in protoscoleces cultured into the metacestode direction. The aim of the present study was to explore how far different in vitro generated stage-specific materials/structures match the natural situation on the transcriptome level, based on testing five exemplarily chosen different genes: the frizzled receptor *eg-fz4* (posterior marker), the FGF receptor-like factor *eg-fgfrl* (anterior association), the cell differentiation protein *eg-rcd1* (part of the CCR4-NOT complex, a key regulator of eukaryotic gene expression), the rapidly accelerated fibrosarcoma serin/threonin kinase *eg-braf* (part of the MAPK pathway involved, e.g., in EGF signaling) and the co-smad *eg-smadD* (downstream factor of TGFβ/BMP2/activin signaling). These genes—tested via qPCR—were selected such as to allow a discussion on their potential role in the development of *E. granulosus* into the adult stage. Thus, testing took place with three ex vivo isolated samples, namely (i) egg-containing adult worms, (ii) invaginated protoscoleces, and (iii) protoscolex-free germinal layer tissue. Respective data were compared (a) with in vitro generated metacestode-like microcysts developed from protoscolices, and (b) different development stages of protoscoleces in vitro cultured toward adult maturation. As a finding, only *eg-smadD* and partially *eg-fz4* showed high expression similarities between ex vivo harvested and in vitro cultured *E. granulosus*, thus suggesting a putative role in adult maturation. Conclusively, the fact of using “only” five genes did not allow answering the question if ex vivo and in vitro materials are similar on the transcriptome level. Another experimental restriction arises from different growth conditions of the in vitro cultured materials, and comparing these to the ex vivo harvested ones. Future experiments may solve the problems by using fully standardized *E. granulosus* sample collection and fully standardized culture conditions.

## Introduction

*Echinococcus granulosus* sensu lato is a group of cestode tapeworms that act as the causative agents of cystic echinococcosis (CE), one of the main neglected tropical diseases recently considered by the World Health Organization (WHO) (da Silva 2010; WHO 2015). As a major public health concern, the disease affects primarily humans, while in domestic livestock, echinococcosis represents predominantly an economic problem. The main definitive host within the life cycle of the parasite is the dog, where adult tapeworms attach to the intestinal epithelium and undergo sexual reproduction, leading to the production of parasite eggs. These eggs are shed into the environment via feces. The egg contains an oncosphere, which hatches in the small intestine of an intermediate host, and subsequently migrates via the portal system to the liver or other organs, where it develops into a fluid-filled metacestode (hydatid cyst) that internally produces protoscoleces. The definitive host becomes infected through the consumption of viscera of intermediate hosts that harbors fertile metacestode forms (“hydatid cysts”) containing protoscoleces (Thompson and Jenkins 2014; Thompson and Lymbery 1995).

Parts of the natural life cycle can be retracted in vitro such as the development of protoscoleces into multiproglottid adult stages, when cultured in an appropriate diphasic medium. However, egg production in the terminal proglottid could not be observed under these conditions until now (Hijjawi et al. 1992). A reason for that observation might be missing dog-intestine derived triggers during in vitro culture, since egg-free young adults extracted from dog and transferred to similar in vitro cultures develop eggs (Kumaratilake et al., 1983). Also, metacestode- or cyst-like structures can be generated during in vitro culture of protoscoleces using monophasic medium conditions (Rogan and Richards 1986). The capability of the protoscoleces to differentiate depending on the culture conditions in vitro into adult-like or metacestode/cyst-like stages allows studying the unusual and unique developmental plasticity of *Echinococcus*. However, until now, it is not known how exact the in vitro generated material reflects the natural situation/reality.

To answer this question, on an exploratory level, we compared the expression of five genes representing different pathways that are known to be associated in maturation and/or stage-specific development in free-living worms such as planarians or *Caenorhabditis elegans* or helminths such as *Schistosoma* species (Saberi et al. 2016; Molin and Puisieux 2005; Scimone et al. 2016; Morel et al. 2014; Andrade et al. 2014).

Since it was shown that the metacestode larval stage of the closely related fox-tapeworm *E. multilocularis* consists only of posterior tissue (Koziol et al. 2016), we chose two anterior/posterior-associated genes. First, the *E. granulosus* frizzled receptor *eg-fz4*, a homologue of the *E. multilocularis* frizzled receptor *em-fz4* described as a posterior marker in protoscoleces (Koziol et al. 2016) and secondly, a C-terminal truncated FGF receptor-like factor (*eg-fgfrl*) whose homologues are anterior markers in planarians (Scimone *et al*., 2016). The three other genes were chosen to cover different developmentally important pathways. First is the *eg-rcd1*, the *E. granulosus* homologue of the cell differentiation protein rcd1. Rcd1 is a part of the CCR4-NOT complex that is a key regulator of eukaryotic gene expression, plays a role in cell differentiation, and is for example involved in messenger RNA (mRNA) degradation, miRNA-mediated repression, transcription regulation, and nuclear hormone signaling (Collart 2016; Garapaty et al. 2008). Another central pathway involved in eukaryotic development, cell proliferation, apoptosis, and embryogenesis is the MAPK/ERK pathway where extracellular receptor kinases such as EGF-receptors bind their ligands and start a signaling cascade via different intracellular factors leading in gene transcription (Rubinfeld and Seger 2005). Parts of this pathway are described for *E. multilocularis* (Spiliotis et al. 2005, 2006, Gelmedin et al. 2010), and for covering the MAPK pathway in our study, we chose the *E. multilocularis em-braf* homologue from *E. granulosus*: *eg-braf*. Third, to cover Tgf-β, Bmp, and activin signaling that is for example involved in embryogenesis, apoptosis, cell differentiation, and/or cell growth (Horbelt et al. 2012), we chose with *eg-smadD* an intracellular factor that acts downstream of the transmembrane receptors. SmadD is a co-Smad and was described for *E. multilocularis* earlier (Epping and Brehm 2011).

The mRNA expression levels of the five mentioned genes were tested in the ex vivo materials “germinal layer without protoscoleces,” “invaginated protoscoleces,” and “adult worms containing eggs” in comparison to in vitro generated “protoscolex-derived microcysts” or “metacestode-like structures” and different stages of “protoscoleces developing toward adult stage.”

## Materials and methods

### Ethics statement

This study was carried out in accordance to protocols approved by the Research Ethical Review Committee of Kerman University of Medical Science (Permit Number 92/274, 2013). Moreover, all efforts were made to minimize suffering of the dog experimentally infected with *E. granulosus*.

### Sample collection

Hydatid cysts were collected from livers of naturally infected sheep at Kerman abattoir, southeastern Iran. Fresh cysts were immediately transferred to the Parasitology Laboratory at the Department of Parasitology, Kerman University of Medical Sciences, Kerman, Iran. After aspiration of hydatid fluid from a cyst by use of a 20-mL syringe and an 18-G needle, the cyst was aseptically opened and the laminated/germinal layer was removed. The layer was carefully washed four times with sterile PBS and microscopically observed to make sure that it is protoscolex-free. The parasite material was then stored at −80 °C, until testing. The hydatid cyst fluid was aseptically transferred into a flask and subsequently left for 30 min to allow protoscoleces to sediment. The supernatant was discarded, and the protoscoleces were washed three times with PBS (pH 7.4). The number of protoscoleces was adjusted to 2 × 10^3^ protoscoleces per milliliter 0.9 % NaCl solution with a viability rate of at least 90 %. The viability of the protoscoleces was confirmed by their flame cell motility and impermeability to 0.1 % eosin solution under a light microscope (Brehm and Spiliotis 2008; Smyth 1962, 1971). The obtained protoscoleces were activated by digestion in 1 % (*w*/*v*) pepsin prepared in 0.85 % (*w*/*v*) sodium chloride, pH 4.0 (adjusted with 2 M HCl) for 30 min at 37 °C to release protoscoleces from capsules; subsequently, the protoscoleces were used for three different purposes: (i) in vitro culture in diphasic media to reach the adult form, (ii) in vitro culture with feeder cells and formation of cystic metacestode stage, and (iii) in vivo assay: experimental infection in dog as definitive host.

### Genotyping using cox1 region

Partial mitochondrial DNA region within *cox1* gene was amplified to reveal the genotype of protoscoleces isolated form hydatid cysts by respective sequencing of PCR products. PCR was basically carried out as described by Bowles et al. (1992) using the *cox1* primers JB3 (5′-TTTTTTGGGCATCCTGAGGTTTAT-3′) and JB4.5 (5′-TAAAGAAAGAACATAATGAAAATG-3′). The total genomic DNA was extracted using a DNeasy Blood &Tissue kit (QIAGEN, Hilden, Germany) according to manufacturer’s instructions. The nucleotide sequences obtained were aligned with existing sequences of the known genotypes in the GenBank databases using BLAST program at the National Center for Biotechnology Information (NCBI). Sequence alignment was compared with the previously reported reference sequences of *E. granulosus* and showed 100 % identity with the *E. granulosus* G1 NCBI GenBank sequence HM563011.

### In vitro cultivation of protoscoleces and development into the adult worm

In vitro cultivation of protoscoleces to reach *E. granulosus* adult worms in biphasic medium was performed according to the method described by Smyth and Davies (1974), Smyth (1962), and Smyth (1971), with a few modifications. Briefly, protoscoleces were pepsin-digested to release protoscoleces from capsules. The culture medium was S.10E.H, which was consisted of two phases: (i) liquid phase, containing 260 mL of CMRL 1066 medium (Thermo Fisher Scientific, USA), 100 mL of heat-inactivated fetal calf serum (FCS), 36 mL of 5 % yeast extract (in CMRL 1066), 5.6 mL of 30 % glucose (in distilled water), 1.4 mL of 5 % dog bile in PBS, 20 mM HEPES, 10 mM NaHCO_3_ supplemented with penicillin (100 IU/mL), streptomycin (100 mg/mL), and (ii) solid phase, bovine serum which was coagulated at 76 °C for 20–30 min (Smyth and Howkins 1966; Smyth and Davies 1974; Brehm and Spiliotis 2008; Smyth 1962). Different stages in culture medium were isolated based on the morphological classification described by Smyth et al. (1967). Isolated stages were subsequently stored in RNAlater at −80 °C until RNA extraction (Table 1).

**Table 1:** Timeline of in vitro developmental stages of *Echinococcus granulosus* (G1)

Stages	Morphology	Time (day)
Pre-segmentation	Invaginated protoscoleces (a)	0
	Evaginated protoscoleces (b)	0–1
	Excretory canals and bladder formation (c)	5–7
Segmentation	First proglottid formation (d)	18–22
	Second proglottid formation (e)	26–32
	Testes and genital pore appearance (f)	34–36
	Third or more proglottid (g)	40–55

### In vitro cultivation of protoscoleces and development into metacestode

In vitro culture of protoscoleces into metacestodes was carried out as previously described elsewhere (Brehm and Spiliotis 2008; Hemphill et al. 2002). Initially, protoscoleces were washed three times in Hanks balanced salt solution (HBSS) and were subsequently added into Dulbecco’s minimal essential medium (DMEM), 2 mM glutamine, penicillin (100 IU/mL), streptomycin (100 mg/mL) supplemented with 10 % heat-inactivated FCS. For feeding protoscoleces during subsequent passages, 1 × 10^6^ murine Hepa 1-6 (ATCC® CRL-1830™) were added to a 200-mL flask. Flasks containing protoscoleces were placed in an upright position in an incubator at 37 °C with 5 % CO_2_. The medium was changed every 4 to 8 days.

### Experimental infection of definitive hosts—in vivo development of *E. granulosus* adult worms

In order to establish the experimental infection in dogs, one healthy male mixed breed dog (6 months old) was obtained from a local supplier in Kerman, Iran. The animal was treated with praziquantel (10 mg/kg body weight) prior to the study beginning and kept in an individual cage to allow it to adapt to the living environment and diet. The animal was fed on commercial dog food and water ad libitum. After antihelminthic therapy and subsequent fecal microscopic examination to confirm a negative coprological status, the dog received orally about 10,000 viable protoscoleces of *E. granulosus* with a meal, and all subsequent food (administration once daily) was previously heat-inactivated as described earlier (Jenkins et al. 2000; Rossi et al. 2012).

### Isolation of *E. granulosus* adult worms

Forty-five days post-infection, the dog was euthanized by intravenous administration of a barbiturate overdose (Thiopental Nesdonal® Biochemie GmbH, Austria), and the animal was conventionally necropsied to isolate *E. granulosus* adult worms. For this, the small intestine was removed and then longitudinally opened prior to immersion into warm physiological saline solution. Detached worms together with the intestinal content were passed through sieves of several mesh sizes (1000 to 2000 μm), then the collected and purified worms were washed three times with sterile PBS and kept in RNAlater at −80 °C until RNA extraction (Jenkins et al. 2000; Rossi et al. 2012).

### mRNA expression study by RT-qPCR

#### RNA extraction

From parasites cultured in vitro in biphasic medium, total RNAs were isolated for the different developmental stages: (a) evaginated protoscoleces, (b) juvenile worms with excretory canals and bladder formation, (c) juvenile worms with first proglottid formation, (d) juvenile worms with second proglottid formation, (e) juvenile worms with testes and genital pore appearance, and (f) juvenile worms with third or more proglottid formation (prior to embryonated egg production). Additionally, total RNAs were extracted from (g) microcysts cultured in the presence of hepatoma cells in monophasic medium, as well as from the three ex vivo harvested: (h) invaginated protoscoleces, (i) germinal layer of hydatid cyst from infected sheep, and (j) adult worms isolated from the experimentally infected dog. Total RNAs from these various stages were isolated using RNeasy Mini kit (QIAGEN, Hilden, Germany). Contaminating genomic DNA was removed by in column DNAse digestion according to the manufacturer’s protocol. The quantity and quality of the RNA samples were assessed by NanoDrop ND-1000 spectrophotometer (NanoDrop Technologies, USA). Samples with concentrations above 100 ng/μL, A260/A280 ratios between 1.8 and 2.0, and A260/A230 ratios between 1.7 and 2.0 were retained.

#### cDNA synthesis

For each sample, 1 μg of total RNA was used to synthesize the first strand complementary DNA (cDNA) using Omniscript® Reverse Transcriptase Kit (QIAGEN, Hilden, Germany) with appropriate random primers (Sigma-Aldrich, St Louis, MO, USA) in a final volume of 20 μL according to the manufacturer’s instructions. The final cDNA products were diluted 50 times with nuclease-free water before qPCR.

#### Gene selection and primer design

We chose five developmentally important genes based on the current knowledge on their function; frizzled receptor (*eg-fzd*, GeneDB no. EgrG_000636500), FGF receptor-like factor (*eg-fgfrl*, GeneDB no. EgrG_000842900), cell differentiation protein rcd1 (*eg-rcd1*, GeneDB no. EgrG_000966200), b-raf (*eg-braf*, GeneDB no. EgrG_001079900), and smadD (*eg-smadD*, GeneDB no. EgrG_000217400). The reference genes TATA-box binding protein (*eg-tbp*, GeneDB no. EgrG_000972300), elongation factor 1 alpha (*eg-ef1α*, GeneDB no. EgrG_000982200), and cyclophilin (*eg-cyp*, GeneDB no. EgrG_000920600) were adapted from Espínola et al. (2014). Table 2 summarizes the characteristics of the primers.

**Table 2:** RT-qPCR amplification data of five developmentally important genes in *Echinococcus granulosus* sensu stricto (G1) and the reference genes

Gene name (GeneDB prediction)	GeneDB model no.	Primer: forward/reverse Amplicon size/qPCR Tm
*Eg-fz4* Frizzled (FZD.1)	EgrG_000636500	5′-TCGGTCGCTACAAACGGG/ 5′-GCTGACTGATTCAACGCCC 206 bp/58 °C
*Eg-fgfrl* FGF receptor-like (FGFR)	EgrG_000842900	5′-TCCTGCCGTCAGTACTCC/ 5′-GCGTGGATAACTGTTCTCC 216 bp/58 °C
*eg-rcd1* Cell diferentiation protein (rcd1)	EgrG_000966200	5′-TGCCATACCTATGAGCGATTC/ 5′-CATCTACTAGCTGCGCTGC 208 bp/58 °C
*Eg-smadD* Co-smad (Smad4)	EgrG_000217400	5′-GGGCACACATTCTGCTCC/ 5′-TCCATTGTTGGTGCCGCC 215 bp/58 °C
*eg-braf* B-Raf (Raf)	EgrG_001079900	5′-AAGGCGTTCAAGAATGAGGT/ 5′-GTGTAGGTAATCCATGCCC 218 bp/58 °C
*eg-tbp* TATA-box binding protein (tbp)	EgrG_000972300	5′-TTCCAGCGCTCAGGCACACA/ 5′-CGTGCGCTTTGAGCTATCCGTCT 165 bp/58 °C
*eg-ef1α* Elongation factor 1α (Ef1 α)	EgrG_000982200	5′-TTTGAGAAAGAGGCGGCTGAGATG/ 5′-TAATAAAGTCACGATGACCGGGCG 174 bp/58 °C
*eg-cyp* Cyclophilin (Cyclophilin)	EgrG_000920600	5′-CGACATCTCCATTGGCGGTAAGC/ 5′-TTGTATCCGAAACCCTTCTCACCG 120 bp/58 °C

#### qPCR analysis

Relative quantification of gene expression levels was carried out by using SYBR green PCR Master Mix (QIAGEN, Hilden, Germany) with 7.5 μL of 1:50 diluted cDNA as template per 15 μL reaction; PCR was performed on a Rotor-Gene Q (QIAGEN, Hilden, Germany) according to the manufacturer’s instructions. Amplification conditions were as follows: 95 °C for 15 min, 45 cycles of 94 °C for 10 s, 58 °C for 15 s, and 72 °C for 35 s. Melt curve of each amplicon was analyzed following amplification cycles with ramping increments of 0.1 °C/s from 55 to 99 °C. To determine the PCR amplification efficiency for each candidate gene, standard cDNA dilutions were prepared using five twofold serial dilutions. PCR results were normalized to the levels of *TATA-box binding protei*n (*eg*-*tbp*), *elongation factor 1 alpha* (*eg-ef1a*), and *cyclophilin* (*eg-cyp1*) genes as reference genes (Espínola et al. 2014). The expression level of all selected genes was evaluated using ΔΔCT method. Four technical replicates per biological stage for each gene were performed. A reverse transcription negative control (without reverse transcriptase) for each synthesized cDNA and a nontemplate negative control for each amplicon were included to confirm the absence of DNA contamination.

#### Statistical analyses

Expression levels of different stages were compared with evaginated protoscolex which is the point of in vitro bidirectional development. Data analysis was carried out using SPSS package version 17.0 (SPSS Inc., Chicago, IL, USA). One-way ANOVA test was used to assess the differences between the relative quantities of each gene. *P* < 0.05 was considered as statistically significant.

## Results

### Genotyping using cox1 region

The amplification of partial *cox1* sequence yielded a product of 366 bp. Nucleotide sequences were shown to be *E. granulosus* sensu stricto (G1 genotype) with 100 % identity to GenBank HM563011.

### In vitro cultivation and in vivo development of adult worm

**Fig. 1:**
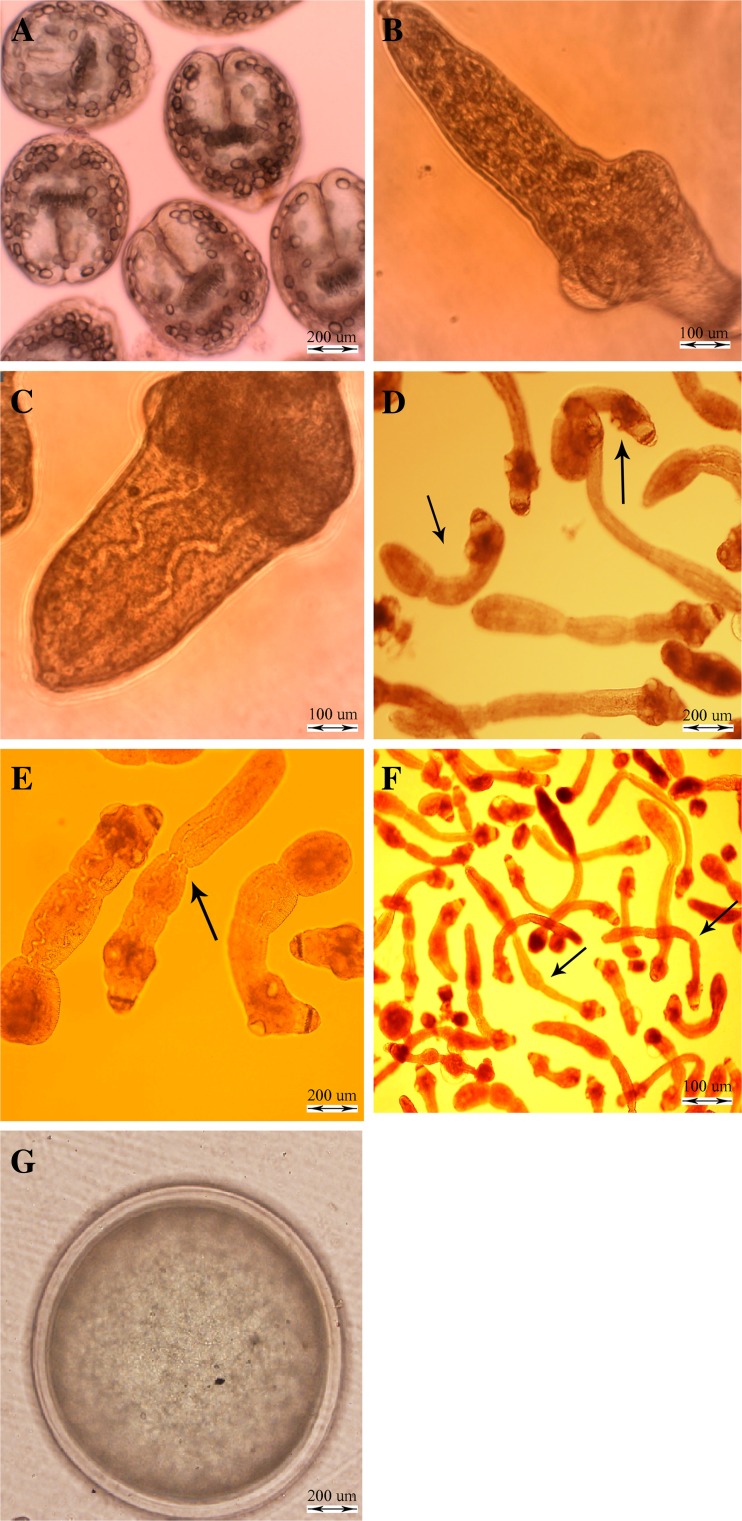
In vitro developmental stages of *Echinococcus granulosus* in monophasic and biphasic culture media: **a** invaginated protoscolex; **b** evaginated protoscolex; **c** excretory canals and bladder formation; **d** first proglottid formation; **e** second proglottid formation; **f** third or more proglottid formation; **g** protoscoleces developed into vesicles after in vitro culture for 6 weeks

Table 1 shows the developmental stages of *E. granulosus* in biphasic culture based on the size and time of development. The different stages of *E. granulosus* in biphasic culture medium were observed as follows: invaginated protoscoleces (Fig. 1a), evaginated protoscoleces (Fig. 1b), excretory canal and bladder formation (Fig. 1c), first proglottid formation (Fig. 1d), second proglottid formation, testes, and genital pore appearance (Fig. 1e), and three or more proglottid formation (Fig. 1f). Figure 1g shows parasite metacestodes after 6 weeks of cultivation of protoscoleces in monophasic culture medium with feeder cells. The cultures were characterized by extensive proliferation of parasite vesicles (metacestodes), without the formation of new protoscoleces. The mature adult worms of *E. granulosus* with three proglottids (not shown) were isolated and subsequently purified from the small intestine of experimentally infected dogs 45 days post-infection.

### Analysis of gene expression levels at different developmental stages

**Fig. 2:**
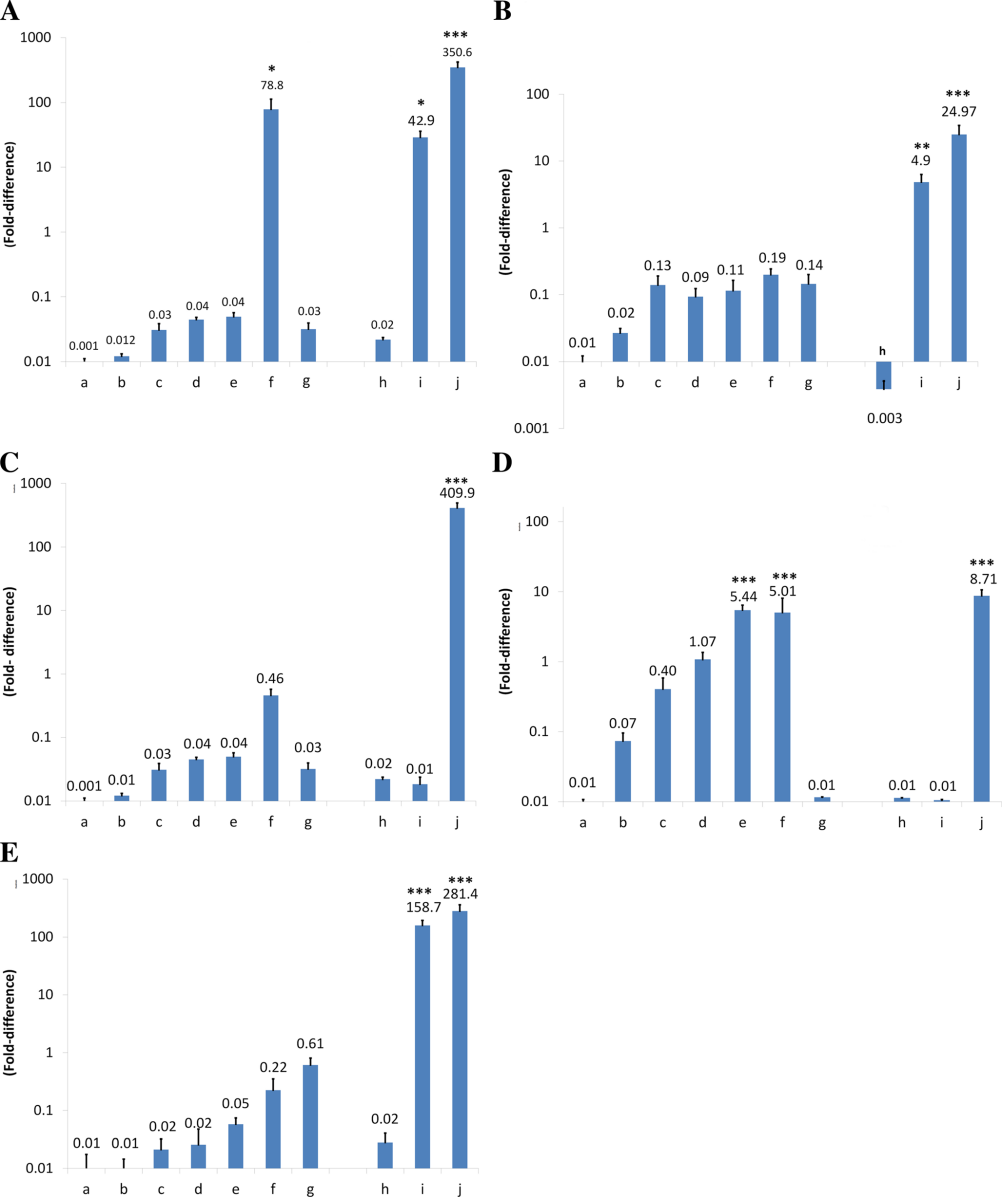
Expression levels of five different, developmentally important genes, i.e., **a** *eg-fz4*, **b** *eg-fgfrl*, **c** *eg-rcd1*, **d** *eg-smadD*, and **e** *eg-braf*, at different developmental stages of *E. granulosus*. Values were all normalized to the evaginated protoscolex. Evaginated protoscoleces (*a*); excretory canal and bladder formation (*b*); first proglottid formation (*c*); second proglottid formation (*d*); testes and genital pore appearance (*e*); third or more proglottid (*f*); microcysts in monophasic media (*g*); invaginated protoscoleces (*h*); germinal layer of hydatid cyst (*i*); adult worms isolated from dog (*j*)

Figure 2 shows the expression levels of the five genes *eg-fz4*, *eg-fgfrl*, *eg-rcd1*, *eg-braf*, and *eg-smadD* in the different in vivo and in vitro developmental stages of *E. granulosus*. The in vitro development of protoscoleces from evaginated protoscoleces to multi-proglottid adult-like forms is shown in bars a–f. In vitro generated metacestode-like forms are shown in bar g. The ex vivo harvested materials from cysts are invaginated protoscoleces (h) and protoscolex-free germinal layer (i) and the ex vivo harvested adult worms from an infected dog is shown in bar j. All expressions were compared to the level of evaginated protoscoleces (a).

To speculate about the questions if the in vitro generated material reflects the natural situation in case of adult development, the expression levels of stages f and j were compared (Q1) and in case of the metacestode/cyst, stages g and i were compared (Q2).

To investigate if time-dependent gene expression levels correlate with adult worm development, we compared different stages of protoscoleces (a–f) cultured in vitro in diphasic medium in view of deregulated gene expression and increasing time (Q3). The question if one of the assessed genes could be involved in sexual maturation and/or egg development, we compared expression levels of the *in vitro* generated semi-adult stage (e, f) with the adults harvested from dogs (j). A deregulated expression between both samples could be a sign of importance for sexual maturation and/or egg production (Q4). To test if one of the genes could be involved in protoscolex development, the expression levels of in vitro cultured dedifferentiated protoscoleces (g) was compared with the ex vivo harvested cyst derived germinal cells (i) (Q5). In the following, the results are separately presented for each gene:

- Frizzled *eg-fz4* (Fig. 2a): Compared to the evaginated protoscoleces (a), *eg-fz4* is highly expressed in the in vitro cultured protoscoleces at the “three or more proglottid” stage (f) (*P* < 0.05) and the two ex vivo materials “germinal layer” (i) (*P* < 0.05) and the “adult worm” harvested from dog (h). All other samples showed low expression; **Q1**: *eg-fz4* was highly expressed in both in vitro developed adult-like stages (f) and the ex vivo harvested adults (j); **Q2**: *eg-fz4* was low expressed in the in vivo cultured metacestode-like structures (g) and highly expressed in the native in vivo grown cyst derived germinal layer (i); **Q3**: during in vitro culture and development of protoscoleces, the expression of *eg-fz4* was very low up to the point when three or more proglottids were formed (a–f), and the expression level finally raised to a very high value (f); **Q4**: during in vitro development from protoscoleces to semi-adults, the expression of *eg-fz4* was peculiarly popping up to a very high level at the time point when worms started to contained genital pores (i) and worms that formed three or more proglottids; **Q5**: *eg-fz4* was low-expressed in in vitro generated microcysts (g) and highly expressed in the ex vivo harvested germinal tissue.
- FGF receptor-like *eg-fgfrl* (Fig. 2b): *eg-fgfrl* was highly expressed in the two ex vivo parasite structures “germinal layer” (i) (*P* < 0.01) and the “adult worm” harvested from dog (h) (*P* < 0.001). All other samples exhibited a low expression level. **Q1**: *eg-fgfrl* was differentially expressed between in vitro developed adult-like stages (f) and the ex vivo harvested adults (j) showing a 131 times higher expression level in the ex vivo harvested adults (j) (*P* < 0.001). **Q2**: A similar differential expression was observed between the in vivo cultured metacestode-like structures (g) and the native in vivo grown cyst derived germinal layer (i) with 35 times higher expression levels in the latter one (*P* < 0.05). **Q3**: Low increase of *eg-fgfrl* expression started in protoscoleces that developed excretory channels and bladder formation (b), while expression remained stable and relatively low throughout the whole in vitro-cultured protoscolece phase (a–f). **Q4**: *eg-fgfrl* was 131 times higher expressed in the ex vivo harvested adults (j) than in the in vitro developed adult-like stages (f). **Q5**: *eg-fgfrl* was 35 times higher expressed in the ex vivo harvested germinal tissue (i) than in the in vitro generated microcysts.
- Cell differentiation protein eg-*rcd1* (Fig. 2c): A very high expression level of *eg-rcd1* was only observed in ex vivo harvested adult worms (j) (*P* < 0.001). All other samples showed low expression. **Q1**: *eg-rcd1* was differentially expressed between in vitro-developed adult-like stages (f) and the ex vivo harvested adults (j), showing a 891 times higher expression level in the ex vivo harvested adults (j) (*P* < 0.001). **Q2**: Between in vivo-cultured metacestode-like structures (g) and native in vivo-grown cyst-derived germinal layer (i), a similar and very low expression level of *eg-rcd1* was observed. **Q3**: A relative constant and low expression level of *eg-rcd1* was notified between the temporally different in vitro-cultured developing protoscolex samples (a–e), and a slightly higher expression was detected in the “three or more proglottid” stage (f). **Q4**: *eg-rcd1* was 891-times higher expressed in the ex vivo harvested adults (j) than in the in vitro developed adult-like stages (f). **Q5**: *eg-rcd1* was at an almost similar low expression level in the ex vivo harvested germinal tissue (i) and in in vitro generated microcysts (g).
- smadD *eg-smadD* (Fig. 2d): *eg-smadD* was minimally expressed in the in vitro cultured and the ex vivo harvested metacestode/cyst materials (g/i) as well as in the invaginated (h) and evaginated (a) protoscoleces. The expression of *eg-smadD* raised by time during in vitro cultivation of protoscoleces (b–d), up to a very high level in e and f, which was directly comparable to the level observed in ex vivo harvested adult worms of dog origin (h) (*P* < 0.001). **Q1**: *eg-smadD* showed a similar high expression in the in vitro cultured “third or more proglottid” semi-adults (f) and the adults harvested from a dog. **Q2**: Minimal expression of *eg-smadD* was measured in both, the in vitro generated metacestode structures (g) and the ex vivo harvested germinal layer material (i). **Q3**: A developmental stage-dependent increase of *eg-smadD* expression was detected in the adult-directed in vitro cultured protoscoleces (a–f), leading to similar expression levels as measured in the ex vivo harvested adults (j). **Q4**: *eg-smadD* was 1.73 times higher expressed in the ex vivo harvested adults (j) than in the in vitro developed adult-like stages (f). **Q5**: *eg-smadD* was at an almost similarly low expression level in both, the ex vivo harvested germinal tissue (i) and the in vitro generated microcysts (g), respectively.
- braf *eg-braf* (Fig. 2e): The expression of *eg-braf* showed very high expression levels in the two ex vivo harvested materials “germinal layer” (i) (*P* < 0.001) and the “adult worms” of dog origin (h) (*P* < 0.001). All other samples showed low expression levels. **Q1**: A differential expression was measured between in vitro developed adult-like stages (f) showing low levels, and the ex vivo harvested adults (j) showing 1279 times increased *eg-braf* expression levels. **Q2**: A similar situation was observed with metacestode material, where low expression levels in the in vitro sample (g) and very high expression levels in the ex vivo sample (i) were determined. **Q3**: A slight increase of *eg-braf* expression during protoscolex development was observed (a–f), but the values were small, and persistently more than 1300 times lower than in the dog-derived adult worms (i). **Q4**: *eg-braf* was 1279 times higher expressed in the ex vivo harvested adults (j) than in the in vitro developed adult-like stages (f). **Q5**: *eg-braf* was almost at a similarly low expression level in the ex vivo harvested germinal tissue (i) as it was in the in vitro generated microcysts (g).

## Discussion

The present study was designed to comparatively investigate—at an exploratory level—microscopically detectable similarities between in vitro generated and ex vivo harvested *E. granulosus* cellular structures, and putatively associated gene expression levels, respectively. Such findings would provide a molecular background for addressing the question how far in vitro generated parasite structures mimic the natural in vivo or ex vivo situation, respectively (Q1 and Q2, as mentioned in the “Results” section). A further aim was to investigate if gene expression levels can be correlated with larval stages of different maturity status, or with adult and semi-adult worms (Q3–5). Answers on these questions may yield a solid basis for complex whole transcriptome sequencing follow-up experiments, especially on experimental setup strategies, and possible respective drawbacks.

With regard to the first questions (Q1/2), our results, although obtained with only five investigated genes, clearly demonstrated the occurrence of high variations between most of the in vitro generated and the ex vivo harvested materials. However, only *smadD* showed an expression pattern that actually allowed a clear correlation between the tested samples; a high expression level marked the adult and semi-adult stages on one hand and a very low expression of both metacestode stages on the other hand. The similar expression levels of *smadD* between the in vitro cultured and the ex vivo harvested materials can be interpreted as a reflection of the natural situation. Partial mimicking was found for *eg-fz4*, which was highly expressed in the ex vivo and in vitro adult stages, but differentially expressed in the tested metacestode samples, and for *eg-rcd1* that was very low expressed in both metacestode samples but divergently expressed in the adult samples. All other genes showed expression differences between the analyzed ex vivo and in vitro samples. Therefore, even if some combinations showed high similarities, the gene expression levels of the in vitro generated materials did not perfectly mimic the ones of the natural ex vivo materials. Possible explanations for that resulting conclusion include (i) differences in the complex growth conditions between in vivo and in vitro grown *E. granulosus* materials influencing gene expression intensity and (ii) the choice/selection of the five tested genes may have been biased by reasons yet unknown to us. In the frame of the present exploratory study, experimental setup difficulties or inaccuracies can be detected and used to improve the performance of complex transcriptome sequencing follow-up experiments. For the questions if in vitro-generated *E. granulosus* material mimics the natural situation, a future setup should be planned for example with a more stringent sample classification. We used in vitro generated microcysts derived from protoscoleces that were very young and did not maturate within brood capsules, and compared them with ex vivo harvested germinal cells that were physically isolated from protoscoleces. In that case, we compared two samples of protoscolex-free germinal cells, but the ex vivo ones appeared at a developmentally more advanced stage, explained by the fact that they contained protoscoleces before. On the other hand, de-developed protoscoleces looked microscopically similar to metacestodes, but if they were metacestodes identical to naturally grown ones on the transcriptome level is not clear. Therefore, in future experiments, variability of conditions should be kept as low as possible. Since the *E. granulosus* material that can be cultured in vitro is limited in a way that two main developmental steps cannot be reproduced until now, the development of protoscoleces inside of in vitro cultured cysts and the development of infective eggs in semi-adult worms, ex vivo samples that are similar to the in vitro generated materials have to be chosen for future experiments to answer the mimicking question successfully. Native ex vivo samples that are similar to the in vitro cultured ones could be semi-adult worms without infective eggs isolated from experimentally infected dogs, and nonfertile cysts isolated from natively infected intermediate hosts, or newly developing cysts isolated from experimentally infected intermediate hosts. An additional variable condition that should be omitted is different growth conditions. Therefore, ex vivo harvested material could be cultured for a certain time in vitro prior sample preparation to minimize these effects.

The second aim of this study was to prove if gene expression levels could be correlated with the presently selected larval stages or the adult and semi-adult worms (Q3–5). The five tested genes might not be sufficient in number to substantially argue about their impact or functional role in developmental processes during maturation of adult or protoscolex development in metacestode tissue. However, the question-based interpretation of the results could first show if our experimental setup generated valuable data concerning these questions, and if that is the case, it could secondly be used as a prerequisite for complex whole transcriptome follow-up approaches. Since we do not want to overinterpret our results, we discuss in the following part not every question for every gene, but highlight the interesting findings concerning the questions (Q3–5) and the usefulness of that approach to be used for answering these or similar questions.

One of the main points addressed was if changing gene expression levels do correlate with in vitro developmental features of protoscoleces into semi-adult worms (Q3). For *eg-smadD*, a clear development-dependent increase of expression intensity was detected, with values reaching similar high levels between the semi-adults containing three or more proglottids and the ex vivo harvested adult worms. This indicated that an increasing *eg-smadD* expression could be part of the adult development of *E. granulosus*. Since Smads are downstream factors of TGFβ/BMP/activin signaling, it can be speculated that Tgf-β/Bmp/activin pathways are involved during the adult development. Specific experiments concerning the possible impact of Tgf-β/Bmp/activin signaling during adult development could be performed by adding host or *E. granulosus* cytokines of the Tgf-β/Bmp/activin family or Tgf-β/Bmp/activin pathway-specific inhibitors into the developing cultures.

Regarding gene expression characteristics respective to egg maturation within adult worms (Q4), the expression level of *eg-fz4* was interesting. The gene exhibited a relatively low expression level during the first phase of in vitro development of protoscoleces into adults, but increased rapidly in semi-adults containing three or more proglottids, showing levels that became similar to the *ex vivo* harvested adults. This indicated that *eg-fz4* and Wnt signaling could play a role during sexual maturation and/or egg production of the adult worms. It can be speculated that *eg-fz4* is expressed and waiting for a specific Wnt signal for further development, but the necessary factors are not present in the in vitro system or cannot be produced by the semi-adult worms due to missing triggers. Possible experiments to clarify the importance of *eg-fz4* on egg maturation could be performed with siRNA knockdown experiments (Mizukami et al. 2010). One possibility would, e.g., be the extraction of young and nonfertile adults from definitive hosts, knockdown *eg-fz4* and follow the egg maturation in vitro.

Furthermore, we selected *eg-fz4* as posterior and *eg-fgfrl* as anterior markers to discriminate between posterior development such as described for metacestode material in *E. multilocularis* (Koziol et al. 2016) and anterior material such as strobilar forms of the worm in dependence on development studies in planarians (Scimone et al. 2016). For our setup, both markers failed. We did not observe more *eg-fz4* in de-differentiated protoscoleces compared to protoscoleces cultured into the adult direction. Here, it has to be mentioned that in the *E. multilocularis* study of Koziol et al., *em-fz4* was used for in situ hybridizations as a posterior marker for protoscoleces and not for metacestode tissue. Therefore, it could play its major role in protoscolex/adult development, a hypothesis that is supported by the finding that *eg-fz4* was highly expressed in the ex vivo adult and in vitro semi-adult stages. Using *eg-fgfrl* as anterior marker should allow us to discriminate between protoscolices containing an anterior/posterior axis, and the metacestode stages missing the anterior pole, if they are young and do not contain brood capsules or protoscoleces. In the case of protoscolex de-differentiation, we expected a reduction of *eg-fgfrl* expression, but the amounts determined in the developed microcysts were similar or even slightly higher than those in the protoscoleces developing into adults. A possible explanation for this observation may be that the developed metacestode-like looking protoscoleces still contained an anterior/posterior axis. Since the naturally in vivo-grown cyst, used to isolate protoscoleces, may still have contained adhering germinal tissue, the high expression values of *eg-fgfrl* could be explained with residual anterior tissue contaminating our samples.

Similarly to *eg-smadD*, *eg-fz4*, and *eg-fgfrl*, the other genes could also be intensively discussed in view of the questions (Q3–5), but in our opinion, this may lead to an overinterpretation of respective results, especially as the expression values did not contribute to the explanation regarding the questions of interest for our aims. Therefore, discussion remains restricted to three genes only. Overall, having investigated “only” five genes turned out to be not enough to yield a comprehensive answer for all five questions raised in this paper; thus, it became evident that future experimental setups will require much more genes, such as, e.g., yielded by whole transcriptome analyses.

## Conclusion and outlook

We exploratively selected five exemplary genes to assess them for their relevance regarding sexual maturation and egg development in adult worms. With *eg-smadD* and *eg-fz4* and the associated Tgf-β/Bmp/activin and Wnt pathways, we now present two highly interesting candidates for further and deeper analyses. Additionally, we identified pitfalls concerning the classification of the used samples that could hamper the identification/generation of solid data in future investigations. Taken together, the results of this exploratory study (i) will help us and others in the sample planning process for future similar experiments, (ii) yielded the identification of interesting genes that could be important for adult development and sexual maturation, and, (iii) finally, will help to elaborate future experimental setups that could be used to identify the missing triggers needed for the in vitro development of protoscoleces into microcysts or the in vitro generation of fully mature adults.
